# Probing the Anticancer Action of Novel Ferrocene Analogues of MNK Inhibitors

**DOI:** 10.3390/molecules23092126

**Published:** 2018-08-23

**Authors:** Supojjanee Sansook, Ella Lineham, Storm Hassell-Hart, Graham J. Tizzard, Simon J. Coles, John Spencer, Simon J. Morley

**Affiliations:** 1 Department of Chemistry, School of Life Sciences, University of Sussex, Falmer, Brighton, East Sussex BN1 9QJ, UK; sansook.s@pnu.ac.th (S.S.); S.Hassell-Hart@sussex.ac.uk (S.H.-H.); 2 Faculty of Science and Technology, Princess of Naradhiwas University, Khok Khian 96000, Thailand; 3 Department of Biochemistry, School of Life Sciences, University of Sussex, Falmer, Brighton, East Sussex BN1 9QG, UK; e.lineham@sussex.ac.uk; 4 UK National Crystallography Service, Chemistry, Faculty of Natural and Environmental Sciences, University of Southampton, Southampton SO17 1BJ, UK; Graham.Tizzard@soton.ac.uk (G.J.T.); S.J.Coles@soton.ac.uk (S.J.C.)

**Keywords:** ferrocene, anticancer, MNK, cell viability, spheroids

## Abstract

Two novel ferrocene-containing compounds based upon a known MNK1/2 kinase (MAPK-interacting kinase) inhibitor have been synthesized. The compounds were designed to use the unique shape of ferrocene to exploit a large hydrophobic pocket in MNK1/2 that is only partially occupied by the original compound. Screening of the ferrocene analogues showed that both exhibited potent anticancer effects in several breast cancer and AML (acute myeloid leukemia) cell lines, despite a loss of MNK potency. The most potent ferrocene-based compound **5** was further analysed in vitro in MDA-MB-231 (triple negative breast cancer cells). Dose–response curves of compound **5** for 2D assay and 3D assay generated IC_50_ values (half maximal inhibitory concentration) of 0.55 µM and 1.25 µM, respectively.

## 1. Introduction

Cancer is among the leading cause of mortality worldwide and the number of cases are expected to rise due to increases in life expectancy and the adoption of poor lifestyle choices. Despite significant advances in early screening and targeted treatments, the prognosis for patients with advanced stage cancer remains bleak [1]. This is particularly evident for late-stage breast cancer, of which 15% are classified as triple negative breast cancer (TNBC), defined as tumours that lack oestrogen receptor (ER), progesterone receptor (PR), and human epidermal growth factor receptor 2 (HER2) expression [2]. These cells display highly aggressive clinical behaviour and limited treatment options have led to poor survival rates [3].

One of the major flaws of conventional treatment is the development of drug resistance due to the heterogeneous expression of drug targets within a tumour [4,5]. For example, a HER2^+^ breast cancer classification requires only 30% of the cells to stain positive for HER2 by immunocytochemistry [6]. Targeted treatment eliminates sensitive subclones but allows the growth of resistant subclones and the progression of emergent subclones [5]. Intratumour heterogeneity may be overcome by targeting the process of protein synthesis. Translation occurs in all cells but is significantly increased in cancer cells, which require an elevated level of protein to meet the metabolic demand. This provides a therapeutic window for treatment [7,8].

**Figure 1:**
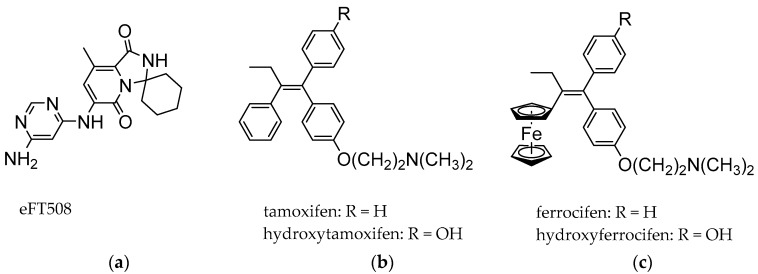
Chemical structures. (**a**) Structure of eFT508, a MNK1/2 inhibitor, (**b**,**c**) structures of prodrugs and hydroxylated drugs; tamoxifen and ferrocifen.

The MNK kinases (MNK1 and MNK2) integrate multiple signals from RAS-RAF-MEK-ERK1/2 (ras/mitogen-activated protein kinase kinase kinase/mitogen-activated protein kinase kinase/mitogen-activated protein kinase) and p38MAPK signalling pathways (mitogen-activated protein kinase) and phosphorylate eukaryotic initiation factor 4E (eIF4E), a key regulator of translation initiation [9]. The phosphorylation of eIF4E (eIF4E-P) is correlated with poor clinical outcome in advanced-stage breast cancer patients and selectively regulates the translation of a subset of mRNA involved in oncogenesis [10,11]. Selective MNK kinase inhibition may limit oncogene-driven signaling [12]. There are currently no FDA-(Food and Drug Administration) approved drugs that specifically act on MNK1/2, although recent progress has been made with the development of eFT508 (Figure 1a), which has a novel pyridone−aminal structure. The molecule eFT508 (Tomivosertib) selectively targets MNK1/2 and is under evaluation in phase II clinical trials for solid tumours and lymphoma [13].

The incorporation of ferrocene into drugs has attracted significant attention in the field of medicinal chemistry, and several ferrocene derivatives have been investigated, including ferroquine and ferrocifen (Figure 1c), which act as antimalarial and anticancer compounds, respectively [14]. Ferrocene is a nontoxic, air- and water-stable complex, comprised of an iron atom sandwiched between two cyclopentadienyl rings [15]. The reversible redox properties of ferrocene have enabled ferrocene derivatives to be used as prodrugs in certain biological environments where ferrocene (Fc) is oxidised to form a cytotoxic ferrocenium cation (Fc+) [14].

Tamoxifen (TAM) is a prodrug that is currently used to treat ER+ breast cancer (Figure 1b). The hydroxylated metabolite, hydroxytamoxifen (OH–TAM), competes with estradiol for binding to ERα and ERβ [16,17]. However, many patients that respond initially to TAM develop resistance to the drug [18]. Ferrocenyl analogues of TAM have been investigated as anticancer agents and have been found to have superior activity against both ER+ and ER- breast cancer cell lines, in contrast to OH–TAM, which is only active against ER+ cells [19]. The replacement of the phenyl group in TAM with a ferrocenyl group led to the formation of the prodrug ferrocifen (Fc–TAM) and the hydroxylated metabolite, hydroxyferrocifen (Fc–OH–TAM), which has an IC_50_ (half maximal inhibitory concentration) of 0.5 µM in TNBC MDA-MB-231 cells [20]. The dual action of Fc–OH–TAM is responsible for its increased anticancer activity. In addition to the competitive binding of ER, Fc–OH–TAM loses two electrons and two protons to form a quinone methide intermediate, a Michael acceptor, capable of instigating a cytotoxic response [21]. Fc–OH–TAM was also found to induce cellular senescence, apoptosis, and the formation of reactive oxygen species (ROS) in a concentration and cell-line dependent manner [22,23,24].

**Figure 2:**
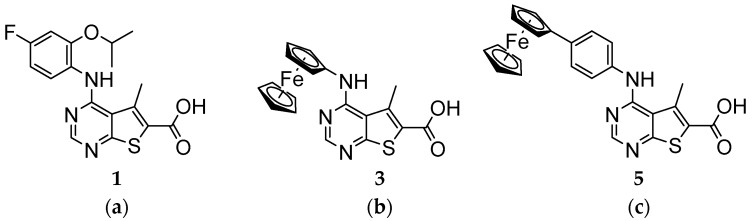
Chemical structures of compounds used in this study. (**a**) Structure of compound **1**, a MNK1/2 inhibitor, (**b**) ferrocene derivative, **3**, (**c**) ferrocene derivative, **5**.

We have previously reported the incorporation of ferrocene, as a phenyl bioisostere, into a variety of different molecules for biological evaluation [25,26,27,28]. From our previous research, we envisaged that the introduction of a ferrocene group to a known MNK1/2 inhibitor (Figure 2a) would both enhance the binding potency and potentially confer other desirable biological properties to the molecules.

## 2. Results

### 2.1. Molecular Modelling of Compound ***1*** Reveals a Large Hydrophobic Cavity in MNK2

**Figure 3:**
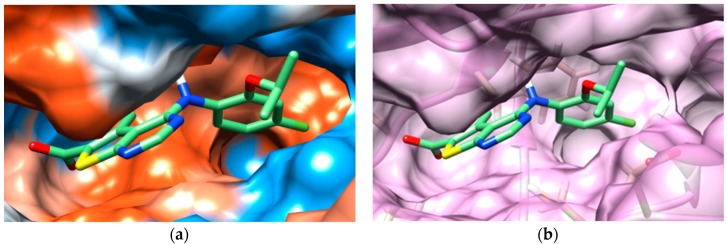
Docking of compound **1** in MNK2 crystal structure. (**a**) Predicted binding mode of compound **1** in MNK2-D228G (PDB; code: 2HW7) coloured by hydrophobicity surface: from blue for the most hydrophilic, to white, to red for the most hydrophobic. (**b**) Important regions within the MNK2 active site and proposed interactions with compound **1**. Molecular graphics and analyses were performed with the UCSF (University of California, San Francisco) Chimera package [33].

The catalytic domains of MNK1 and MNK2 share 78% sequence identity, with active site residues sharing 90% sequence identity [29]. The MNK kinases have unique features that distinguish their structures from other protein kinases. The DFD (Asp-Phe-Asp) motif has less affinity for ATP (adenosine triphosphate) than the equivalent DFG (Asp-Phe-Gly) motif present in other kinases [30]. The crystal structures of MNK1 and MNK2 are available in the DFD-out (inactive) conformation, which blocks ATP-binding [31,32]. A crystal structure of MNK2-D228G in complex with staurosporine (protein databank (PDB): 2HW7) was used to model MNK1/2 inhibitor, **1** (Figure 3a,b). This mutation from wild-type DFD to the canonical DFG motif creates a structure that has both DFG-in and DFG-out conformations that ease crystallisation of the protein [30].

The predicted binding mode of compound **1** in MNK2-D228G indicates that the pyrimidine moiety occupies the ATP-binding pocket, the carboxyl group is solvent exposed, and the fluoroaniline projects into the large hydrophobic pocket. There are a few important interactions to note, depicted in Figure 3b. Firstly, the aromatic side chain of gatekeeper residue, Phe159, provides a surface of negative electrostatic potential that allows the formation of cation-π interactions between the active site and compound **1**. The cations present on the edge of the fluoroaniline ring interact with the negative charge over the face of the benzene ring present in Phe159. There is also a strong sulphur-aromatic interaction between the pre-DFG Cys225 residue and the pyrimidine in compound **1**. Modelling of compound **1** revealed a large hydrophobic pocket, a region that could be exploited by substituting the aniline ring with a bulkier group such as ferrocene.

### 2.2. Synthesis of Ferrocene Analogues

**Scheme 1:**
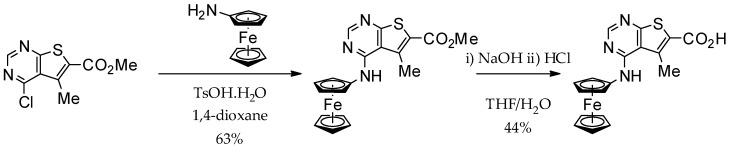
Synthesis of ferrocene analogue, **3**.

**Scheme 2:**
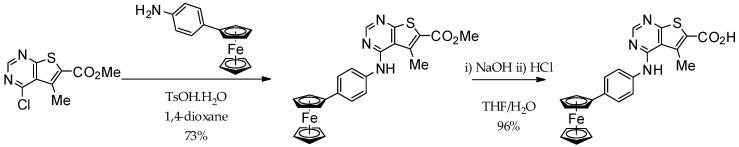
Synthesis of ferrocene analogue, **5**.

**Figure 4:**
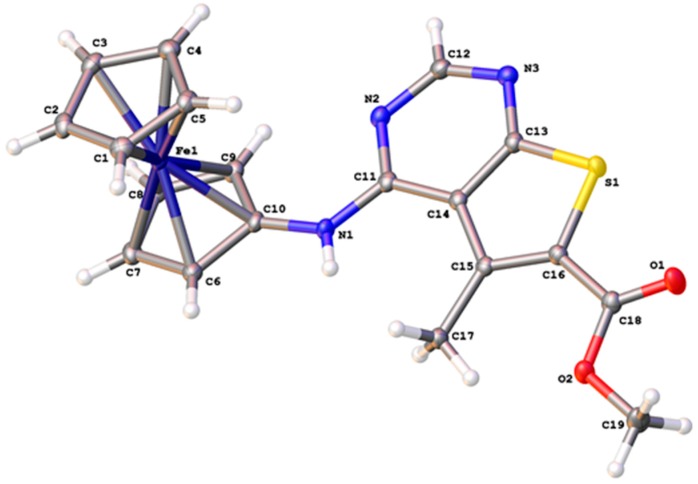
Crystal structure of compound **3** (CCDC 1862154).

The preparation of the ferrocene analogues (**3** and **5**) was conducted using an analogous strategy to the synthesis of **1** (Figure 2a) [34]. In both instances, microwave-mediated acid-catalysed S_N_Ar (nucleophilic aromatic substitution) followed by basic hydrolysis enabled us to synthesise the desired inhibitors in good to excellent yields (Scheme 1 and Scheme 2). A solid-state determination confirmed the structure of **3** (Figure 4). Figures S1–S4 show scanned ^1^H- and ^13^C-NMR spectra of compounds.

### 2.3. In Vitro Analysis of Compound ***1***, ***3***, and ***5*** in Cancer Cell Lines

**Figure 5:**
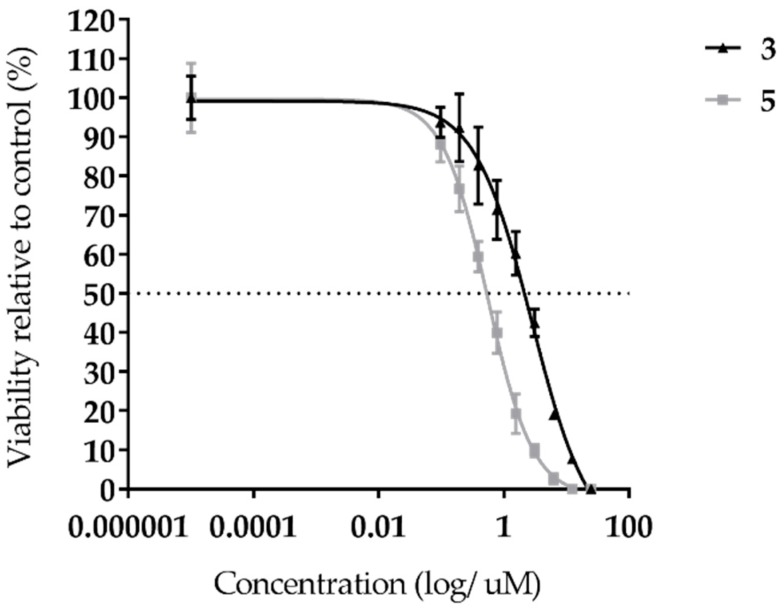
Dose–response curves for ferrocene analogues, **3** and **5** in MDA-MB-231 cells. Cells were treated for 72 h and assessed by Cell Titer Blue assay. Data shown as mean ± standard deviation (SD) of triplicate wells and are representative of three independent experiments (*n* = 3).

Compound **1** had no significant effect on cell viability in any of the three breast cancer cell lines tested, including TNBC cell lines (BT-549 and MDA-MB-231) and the HER2^+^ breast cancer cell line, SK-BR-3 (Table 1). However, **1** did decrease cell viability in MOLM-13 (acute myeloid leukemia) cells and MV-4-11 acute myeloid leukaemia (AML) cell lines, with IC_50_ values of 5.39 µM and 9.72 µM, respectively. The cell viability of compound-**3**-treated cancer cell lines varied according to cell type. Compound **3** appears to have greater potency against BT-549 and MDA-MB-231 cells and is less effective against the HER2^+^ breast cancer cell line, SK-BR-3. The effect of **3** on cell viability in AML cell lines was cell-line-specific, with no significant effect on MOLM-13 and a moderate decrease in cell viability observed in MV-4-11. The most potent compound tested, **5**, showed the highest anticancer activity with an IC_50_ < 2 µM in four of the cell lines tested. Compounds **3** and **5** both showed greater potency towards the TNBC cell line, MDA-MB-231, with IC_50_ values of 2.83 µM and 0.55 µM, respectively (Table 1 and Figure 5). The sensitivity of MDA-MB-231 towards **5** prompted further evaluation and **5** was taken forward for additional studies.

**Figure 6:**
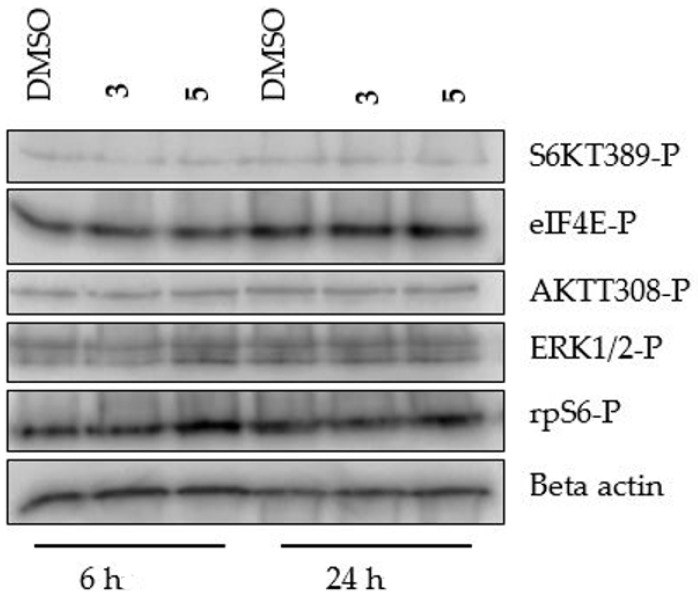
Representative Western blot analysis of both **3**- and **5**-treated MDA-MB-231 cells from three independent experiments (*n* = 3). Western blot analysis showing the effect of 5 μM of **3** and **5** on various signalling molecules after 6 h and 24 h incubation. MDA-MB-231 cells were incubated with DMSO alone (lanes 1 and 4), **3** (lanes 2 and 5) or with **5** (lanes 3 and 6). Cellular lysates were prepared and immunoblotting was performed using 20 μg of total lysate protein, as described in the Materials and Methods.

Compounds **3** and **5** were screened for activity in MDA-MB-231 cells by Western blotting (Figure 6). MNK1/2 both phosphorylate eIF4E on Ser209, enhancing the translation of a subset of mRNA involved in oncogenesis [10,11]. Our data show that incubation of cells with 5 μM of compound **3** or **5** for 6 h had no effect on the level of eIF4E-P. An increase in incubation time to 24 h did not inhibit eIF4E phosphorylation; the slight increase in eIF4E-P observed in all treated cells probably reflects an overall increase in cellular growth. The treatment of MDA-MB-231 cells with **3** and **5** as single agents did not appear to effect PI3-K signalling, indicated by little change in the level of phosphorylation of AKT (T308-P) in comparison to the DMSO (dimethylsulphoxide) control. Phosphorylation of p70S6K (T389) and ERK1/2 (T202/Y204) were also unaffected upon incubation of cells with the ferrocene analogues, **3** and **5**. However, there was a slight increase in the level of ribosomal protein S6 phosphorylation when cells were incubated with **5** at 5 μM, eluding to a possible role of **5** in modulating ribosomal protein S6 phosphorylation.

### 2.4. Treatment of MDA-MB-231 Cells with Compound ***5*** Increases the Rate of Cell Migration

**Figure 7:**
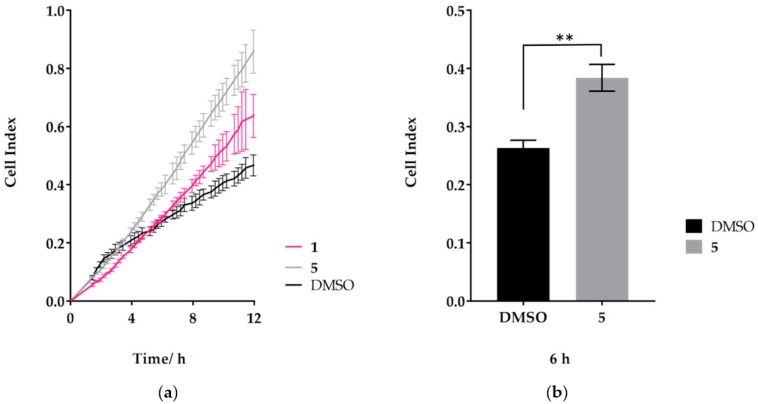
Kinetics of migration in drug-treated MDA-MB-231 cells. Cell migration in real time was analysed by the xCELLigence RTCA. (**a**) Shows the average cell indexes over 12 h for each drug treatment, (**b**) cell migration analysis at 6 h, data shown as mean ± SD of quadruplicate wells and are representative of three independent experiments (*n* = 3); ** *p* ≤ 0.01.

TNBC is an aggressive form of breast cancer typified by highly migratory and invasive cells [35]. Here, we assessed the rate of migration of the TNBC cell line, MDA-MB-231, using real-time monitoring of cell migration (Figure 7). MDA-MB-231 cells were analysed in the presence of DMSO alone, compound **1**, or compound **5** as they moved towards a chemo-attractant. Cell migration kinetics were recorded on an RTCA DP (Real-Time Cell Analyzer Dual-Plate) instrument for 12 h. As shown in Figure 7a, when cells were treated with **1** at 1 μM final concentration, a slight increase in cell migration was observed. The effect of **5** at 1 μM concentration on cell migration was more substantial, with a significant increase in cell migration at 6 h relative to the DMSO control (Figure 7b).

### 2.5. Analysis of Compound ***1*** and ***5*** on MDA-MB-231 Spheroid Growth

**Figure 8:**
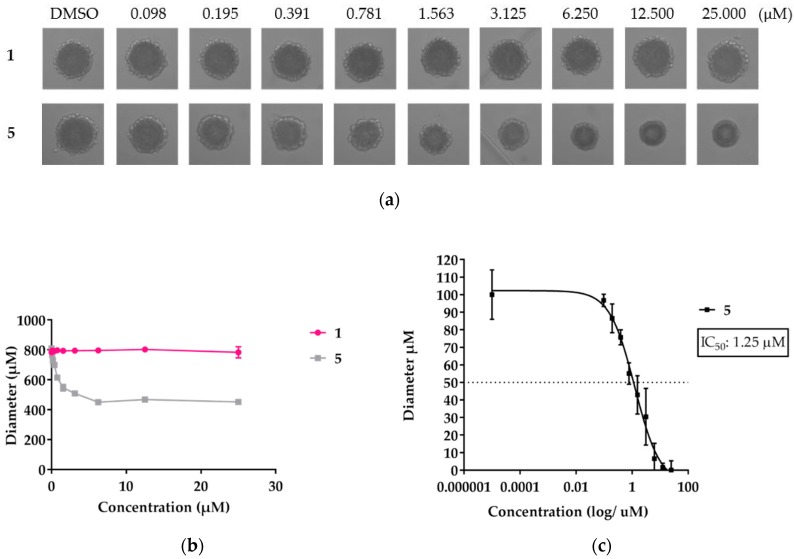
Assessment of spheroid growth on drug-treated MDA-MB-231 spheroids. MDA-MB-231 cells (ER-, PR-, HER2-) were treated for 4 days with **1** and **5** as single-agents. Spheroids were imaged and analysed using the Celigo High Throughput Micro-Well Image Cytometer. (**a**) Representative bright-field images of MDA-MB-231 spheroids after 4 days. (**b**) Diameter of MDA-MB-231 spheroids after 4 days incubation. (**c**) Spheroid diameter dose–response curve for compound-**5**-treated spheroids. Data shown as mean ± SD of triplicate wells and are representative of three independent experiments (*n* = 3).

Next, we examined the sensitivity of MDA-MB-231 cells to compound **1** and **5** in 3D cell culture. Having established the optimal conditions for spheroid assembly (detailed in Materials and Methods), we examined the effect of increasing concentrations of compound **1** and **5** on spheroid growth. Three-day-old spheroids were treated with DMSO alone, compound **1**, or **5** and their growth monitored using the Celigo Image Cytometer. Whole-well bright-field images were acquired multiple days after adding the test compounds and the tumour spheroid diameter was measured to provide a quantitative analysis of spheroid growth (Figure 8a,b). Our results show that compound **1** had no effect on spheroid diameter, even at the highest concentration of 25 µM. In contrast, **5** showed a concentration-dependent growth inhibition of MDA-MB-231 spheroids. A dose–response curve was plotted for **5** and used to calculate an IC_50_ value of 1.25 µM (Figure 8c), approximately twice the IC_50_ value reported in the 2D cell viability assay (IC_50_: 0.55 µM). As with cell growth characteristics, these results indicate that drug susceptibility can vary between 2D and 3D assays and a combination of assays are required to ascertain drug effectiveness.

### 2.6. In Vitro Kinase Assays Report That Compound ***5*** Has No Effect on MNK1/2 Kinase Activity

As compound **3** had poor activity in earlier assays, in vitro target validation of **5**, kinase screening was carried out (commerically, Reaction Biology, Table 2). As expected, compound **1** dramatically reduced MNK1 and MNK2 enzyme activity to ~3% and ~1.5% relative to the DMSO control. Compound **1** had a significant off-target effect on PIM-1, in which enzyme activity was reduced to <50%. This may be explained by the structural similarity between MNK1/2 and PIM-1. The inhibition of MNK1/2 by compound **1** did not affect upstream protein kinases, AKT, and p38MAPK [36]. In contrast, **5** had no effect on percentage (%) enzyme activity in any of the protein kinases screened and an extensive screening panel would be required for more in-depth profiling.

## 3. Discussion

Structural modelling of compound **1** in the active site of MNK kinase revealed a large hydrophobic pocket that could be exploited with a bulkier group such as a ferrocene. Although the ferrocene derivatives, **3** and **5**, showed no activity against MNK1/2, their potency in cancer cell lines is promising. It is unsurprising that compound **1** has no significant effect on cell viability, as the MNK kinases are dispensible for normal development [34]. MNK1/2 inhibitors exert their effect by preventing the drive of oncogenic signalling through the inhibition of eIF4E phosphorylation. The IC_50_ values for the two ferrocene compounds follow a similar pattern across the range of cell lines tested. Both compounds are more effective against TNBC lines and less effective in the HER2^+^ cell line, SK-BR-3. This could reflect a variation in the levels of cellular proteins, or a difference in the rate of metabolism. It is possible that the ferrocene itself is responsible for the anticancer activity. The production of ROS in the Fenton reaction has been proposed as a mechanism of cytotoxicity in other ferrocene derivatives [37]. The anticancer compound, Fc–OH–TAM, elicits an estrogen receptor-independent mechanism of action in addition to its competitive binding of ER. Additionally, ROS production was associated with cell cycle arrest and cellular senescence in Fc–OH–TAM-treated breast cancer cells [24]. The IC_50_ values for compound **5** and Fc–OH–TAM in MDA-MB-231 were both ~0.5 µM, which suggests ROS production may be involved in the mode of action for **5**. The compounds synthesised in this study are currently being investigated to determine if the anticancer effects are due to the generation of ROS.

The synthesised compounds were submitted to a small kinase-screening panel aimed at assessing their ability to inhibit enzyme activity in selected kinases. These data suggest that ferrocene derivatives **3** and **5** target alternative cellular pathways than those investigated. Interestingly, the more potent of the compounds, **5** increased the rate of migration in MDA-MB-231 cells. Compound **5** may target a negative regulator of cell migration; alternatively, the increase in migration rate may be a result of cellular stress. Western blotting also suggested that the level of ribosomal protein S6 (rpS6) phosphorylation increased upon incubation with **5**, indicating a possible role of **5** in the inhibition of protein phosphatases. Further analysis would require an extensive kinase to validate the targets of these compounds. These data, in agreement with the literature, highlight the potential of ferrocene-based compounds as anticancer agents. Whether the ferrocene’s role is via the further generation of reactive oxygen species or a topological effect is under further investigation.

## 4. Materials and Methods

### 4.1. Cell Culture

Breast cancer cell lines BT-549, MDA-MB-231, and SK-BR-3 were sourced from the American Type Culture Collection (ATCC, Manassas, VA, USA), US. The human AML cell lines MOLM-13 and MV4-11 were purchased from the German Collection of Microorganisms and Cell Cultures (DSMZ, Braunschweig, Germany). Cell lines were maintained in Minimal Essential Medium with Glutamax and Earl’s salts (MEM, Gibco, UK), supplemented with 10% (*v*/*v*) foetal bovine serum (FBS, Pan Biotech, Aidenbach, Germany) at 37 °C in a humidified atmosphere with 5% CO_2_.

### 4.2. Cell Viability

To assess cell viability in vitro, all cell lines were seeded into 96-well plates at various cell densities per 100 µL media, then treated with 0.2% DMSO or varying concentrations of compounds for 72 h. Cell viability was measured using the CellTiter-Blue reagent (Promega, Madison, WI, USA) with fluorescence recorded at 560Ex/590Em using a Synergy HT Multi-Detection Reader (BioTek, Winooski, VT, USA).

### 4.3. Cell Migration Assay

Using the xCELLigence DP device from Roche Diagnostics, real-time measurements of cell migration on MDA-MB-231 cells were performed. Cells were seeded at 30,000 per well in CIM-Plates 16 (Roche Diagnostics, Risch-Rotkreuz, Switzerland) in serum-free medium in the presence or absence of inhibitors. Full growth medium was used as a chemo-attractant in the lower chamber. As cells pass through the 8 µm pores towards the chemo-attractant, they adhere to the underside of the filter, embedded with a gold microelectrode. This produces an electrical impedance signal, which correlates with the number of migrating cells. Cell index is an arbitrary unit based upon the measured cell-electrode impedance derived by the software using the following calculation as described in reference [34].

### 4.4. In Vitro Kinase Assays

Compounds were tested as single-dose duplicates at 1 µM by Reaction Biology Kinase HotSpot screening. The enzyme activity (%) of the compound relative to the DMSO control was evaluated in a panel of five kinases.

### 4.5. Three-Dimensional Cell Culture

A centrifugal forced-aggregation method was used to generate MDA-MB-231 spheroids. Briefly, cells were detached from the flasks by trypsin, washed twice with PBS, resuspended, and counted. MDA-MB-231 cells were seeded in Costar Low Attachment U-bottom 96-well plates at 2000 cells/well/100 µL of normal growth media. A 6% Matrigel Matrix (Corning, Corning, NY, USA), media mix was prepared on ice and 100 µL added to each well, to give a final 3% Matrigel Matrix solution. Plates were then centrifuged for 2 min at 1200 g at 4 °C, and incubated for three days at 37 °C, 5% CO_2_. Established spheroids were treated with test compounds at various concentrations and analysed using the Celigo High Throughput Micro-Well Image Cytometer after 4, 6, and 8 days of incubation.

### 4.6. Chemical Synthesis: General Procedures

All reactions were carried out in air using commercial-grade starting materials, solvents, and reagents. The progress of all reactions was monitored by thin layer chromatography (TLC, Silver Spring, MD, USA) using commercially available glass silica gel plates (60 Å, F254). The mobile phase was generally a solvent mixture, and the visualization was undertaken using UV light. All NMR spectra were measured on a Varian NMR 500 spectrometer (Palo Alto, CA, USA) at 500 MHz (^1^H). Chemical shifts are quoted in parts per million (ppm; % relative to a residual solvent peak for ^1^H). Chromatographic purifications were undertaken using an ISCO purification unit, Combi Flash RF 75 PSI, using Biotage silica gel columns. LC-MS (Shimadzu Mass Directed Liquid Chromatography mass spectrometry, Kyoto, Japan) purity analyses were undertaken using a 5 μm C18 110 Å column.

### 4.7. Chemical Synthesis: Experimental

Ferrocenylamine, Methyl 4-chloro-5-methylthieno[2,3-*d*]pyrimidine-6-carboxylate and solvents were purchased from commercial sources and used as such. (4-Aminophenyl)ferrocene was made according to the literature method [38]. High resolution mass spectrometry (HRMS) was performed by the EPSRC National Mass Spectrometry Facility, University of Swansea. Elemental analyses were conducted by Stephen Boyer (London Metropolitan University, Holloway, London, UK). Reactions were carried out in a well-vented fume hood. ^1^H and ^13^C-NMR spectra were recorded on Varian 500 MHz or 400 MHz spectrometers and chemical shifts are reported in ppm, usually referenced to TMS as an internal standard.

*Methyl-5-methyl-4-(ferrocenylamino)thieno[2,3-d]pyrimidine-6-carboxylate* (**2**): To a mixture of methyl 4-chloro-5-methylthieno[2,3-*d*]pyrimidine-6-carboxylate (100 mg, 0.410 mmoL), ferrocenylamine (114 mg, 0.410 mmoL), and *p*-toluenesulfonic acid monohydrate (15 mg, 0.082 mmoL) was added anhydrous 1,4-dioxane (1 mL) under an argon atmosphere. The resulting mixture was heated to 150 °C under microwave irradiation and stirred for 30 min. The resulting mixture was concentrated under reduced pressure. The resulting residue was purified by column chromatography (*n*-hexane/ethyl acetate, 100:00–40:60). The appropriate fractions were combined and concentrated under reduced pressure to give methyl-5-methyl-4-(ferrocenylamino)thieno[2,3-*d*]pyrimidine-6-carboxylate (**2**) as an orange solid (105 mg, 63%). ^1^H NMR (*d*_6_-DMSO, 500 MHz): δ = 8.53 (1H, s), 8.02 (1H, s), 4.82 (2H, s), 4.16 (5H, s), 4.07 (2H, s), 3.84 (3H, s), 3.02 (3H, s). ^13^C NMR (*d*_6_-DMSO, 126 MHz): δ = 171.0, 153.5, 130.1, 125.4, 125.3, 124.3, 124.1, 96.1, 79.8, 69.2, 64.1, 61.1, 36.2, 28.7. HRMS-ESI (*m*/*z*): calc. for [C_19_H_17_FeN_3_O_2_S + H]^+^ = 407.2712, observed = 407.2716. Anal. Calc. (%) for C_19_H_17_FeN_3_O_2_S: C, 56.03; H, 4.21; N, 10.32. Found (%): C, 55.97; H, 4.19; N, 10.21.

*Methyl-4-(ferrocenyl-phenyl-4-ylamino)-5-methylthieno[2,3-d]pyrimidine-6-carboxylate* (**4**): To a mixture of methyl 4-chloro-5-methylthieno[2,3-*d*]pyrimidine-6-carboxylate (140 mg, 0.577 mmoL), 4-(aminophenyl)ferrocene (176 mg, 0.635 mmoL), and *p*-toluenesulfonic acid monohydrate (22 mg, 0.116 mmoL) was added anhydrous 1,4-dioxane (2.5 mL) under an argon atmosphere. The resulting mixture was heated to 150 °C under microwave irradiation and stirred for 30 min. The resulting mixture was concentrated under reduced pressure. The resulting residue was purified by column chromatography (*n*-hexane/ethyl acetate, 100:00–40:60). The appropriate fractions were combined and concentrated under reduced pressure to give methyl-4-(ferrocenyl-phenyl-4-ylamino)-5-methylthieno[2,3-*d*]pyrimidine-6-carboxylate (**4**) as an orange solid (203 mg, 73%). ^1^H NMR (*d*_6_-DMSO, 500 MHz): δ = 8.53 (1H, s), 8.02 (1H, s), 7.52–7.44 (2H, m), 7.01 (2H, d, *J* = 1.9 Hz), 4.82 (2H, s), 4.16 (5H, s), 4.07 (2H, s), 3.84 (3H, s), 3.02 (3H, s). ^13^C NMR (*d*_6_-DMSO, 126 MHz): δ = 171.0, 153.5, 130.1, 125.4, 125.3, 124.3, 124.1, 96.1, 79.8, 69.2, 64.1, 61.1, 36.2, 28.7. ^13^C NMR (*d*_6_-DMSO, 126 MHz): δ = 170.9, 153.0, 130.1, 125.4, 125.3, 124.3, 124.1, 96.1, 80.0, 69.2, 64.1, 61.1, 36.2, 28.7, 28.5, 25.3. HRMS-ESI (*m*/*z*): calc. for [C_25_H_21_FeN_3_O_2_S + H]^+^ = 483.3675, observed = 483.3671. Anal. calc. (%) for C_25_H_21_FeN_3_O_2_S: C, 62.12; H, 4.38; N, 8.69. Found (%): C, 62.04; H, 4.30; N, 8.71.

*5-Methyl-4-(ferrocenylamino)thieno[2,3-d]pyrimidine-6-carboxylic acid:* (**3**)*:* To a mixture of 5-methyl-4-(ferrocenylamino)thieno[2,3-*d*]pyrimidine-6-carboxylate (**2**) (66 mg, 0.162 mmoL), anhydrous THF (5.0 mL), and water (0.5 mL) was added sodium hydroxide (43 mg, 1.080 mmoL). The resulting mixture was heated to 50 °C and stirred for 40 h. The reaction was cooled to ambient temperature and concentrated under reduced pressure. To the residue was added water (10 mL) and the resulting mixture acidified to pH = 4 by the slow addition of conc. aqueous HCl. The resulting mixture was filtered under reduced pressure and washed with water to give 5-methyl-4-(ferrocenylamino)thieno[2,3-*d*]pyrimidine-6-carboxylic acid (**3**) (28 mg, 44%) as an orange solid. ^1^H NMR (*d*_6_-DMSO, 500 MHz): δ = 11.93 (1H, s), 8.50 (1H, s), 8.10 (1H, s), 4.80 (2H, s), 4.16 (5H, s), 4.07 (2H, s), 3.84 (3H, s). ^13^C NMR (*d*_6_-DMSO, 126 MHz): δ = 170.9, 153.5, 130.1, 125.4, 125.3, 124.3, 124.1, 96.1, 79.8, 69.2, 64.1, 61.1, 28.7. IR (cm^−1^): 3339, 1690, 1580, 1005. HRMS-ESI (*m*/*z*): calc. [C_18_H_15_FeN_3_O_2_S + H]^+^ = 393.2415, found = 393.2411. Anal. calc. (%) for C_18_H_15_FeN_3_O_2_S: C, 54.98; H, 3.84; N, 10.69. Found (%): C, 54.91; H, 3.75; N, 10.62.

*4-(Ferrocenyl-phenyl-4-ylamino)-5-methylthieno[2,3-d]pyrimidine-6-carboxylic acid* (**5**): To a mixture of methyl-4-(ferrocenyl-phenyl-4-ylamino)-5-methylthieno[2,3-*d*]pyrimidine-6-carboxylate (**4**) (193 mg, 0.40 mmoL), anhydrous THF (20 mL), and water (5 mL) was added sodium hydroxide (328 mg, 8.20 mmoL). The resulting mixture was heated to 50 °C and stirred for 24 h. The reaction was cooled to ambient temperature and concentrated under reduced pressure. To the residue was added water (10 mL) and the resulting mixture acidified to pH = 4 by the slow addition of conc. aqueous HCl. The resulting mixture was filtered under reduced pressure and washed with water to give 4-(ferrocenyl-phenyl-4-ylamino)-5-methylthieno[2,3-*d*]pyrimidine-6-carboxylic acid (**5**) (180 mg, 96%) as an orange solid. ^1^H NMR (*d*_6_-DMSO, 500 MHz): δ = 11.95 (1H, s), 8.53 (1H, s), 8.02 (1H, s), 7.52–7.44 (2H, m), 7.01 (2H, d, *J* = 1.9 Hz), 4.82 (2H, s), 4.16 (5H, s), 4.07 (2H, s), 3.84 (3H, s). ^13^C NMR (*d*_6_-DMSO, 126 MHz): δ = 170.0, 165.2, 159.1, 156.0, 139.8, 131.8, 127.7, 120.6, 112.2, 110.0, 102.8, 69.4, 68.7, 64.9, 61.7, 28.8, 28.2, 22.4, 14.0. IR (cm^−1^): 3433, 2922, 1682, 1548, 1523, 1495, 818, 752. HRMS-ESI (high resolution mass spectrometry, electrospray ionization) (*m*/*z*) calc. for [C_24_H_19_FeN_3_O_2_S + H]^+^ = 469.3485, found = 469.3484. Anal. calc. (%) for C_24_H_19_FeN_3_O_2_S: C, 61.42; H, 4.08; N, 8.95. Found (%): C, 61.38; H, 3.98; N, 8.92.

### 4.8. Statistics

All statistical analyses were performed using GraphPad Prism 7 software (La Jolla, CA, USA). In vitro log dose–response curves were calculated using nonlinear regression with variable slope after normalizing absorbance to DMSO treated controls, with the concentration required to inhibit the MTS response by 50% reported as the IC_50_.

## Figures and Tables

**Table 1:** Half maximal inhibitory concentration (IC_50_) values of compound **1** vs. ferrocene analogues **3** and **5** in vitro (µM).

Cell Line ^1^	1	3	5
BT-549	n.s	6.67	1.73
MDA-MB-231	n.s	2.83	0.55
SK-BR-3	n.s	114.30	10.63
MOLM-13	5.39	n.s	1.82
MV-4-11	9.72	8.37	1.98

^1^ IC_50_ values are given as mean of three independent experiments (*n* = 3), n.s, nonsignificant.

**Table 2:** Selectivity of compound **1** and **5** against a panel of kinases (% enzyme activity relative to DMSO controls).

Kinase ^1^	1		5	
	Data 1	Data 2	Data 1	Data 2
AKT1	110.03	105.00	106.92	105.28
MNK1	3.64	2.53	115.13	111.38
MNK2	1.87	0.79	97.56	96.04
P38a/MAPK14	125.49	119.86	126.41	125.76
PIM1	43.11	42.99	118.37	117.26

^1^ Compounds were tested in single dose duplicates at 1 μM and reactions were carried out at 10 µM ATP (Reaction Biology Kinase HotSpot Screening).

## Supplementary Materials

The following are available online, Figures S1–S4: scanned ^1^H- and ^13^C-NMR spectra of compounds.
