# Supplementary material for: Probing the Anticancer Action of Novel Ferrocene Analogues of MNK Inhibitors
Source: Molecules. 2018 Aug 23;23(9):2126. doi: 10.3390/molecules23092126 (PMC6225114; doi:10.3390/molecules23092126)
Supplement: Supplementary file 1 [file molecules-23-02126-s001.pdf]

Supplementary Material:

Probing the Anticancer Action of Novel Ferrocene Analogues of MNK Inhibitors.

Supopjjanee Sansook[1,2#], Ella Lineham[3#], Storm Hassell-Hart[1], Graham J. Tizzard[4], Simon J. Coles[4], John Spencer[1*] and Simon J. Morley[3*]

1 Department of Chemistry, School of Life Sciences, University of Sussex, Falmer, Brighton, East Sussex, BN1 9QJ, UK; sansook_s@yahoo.com (S.S.); S.Hassell-Hart@sussex.ac.uk (S.H.H.).
2 Faculty of Science and Technology, Princess of Naradhiwas University, Thailand, 96000.
3 Department of Biochemistry, School of Life Sciences, University of Sussex, Falmer, Brighton, BN1 9QG, UK; e.lineham@sussex.ac.uk (E.L.).
4 UK National Crystallography Service, Chemistry, Faculty of Natural and Environmental Sciences, University of Southampton, Southampton, SO17 1BJ. UK; Graham.Tizzard@soton.ac.uk (G. J. T.); S.J.Coles@soton.ac.uk (S. J. C.).

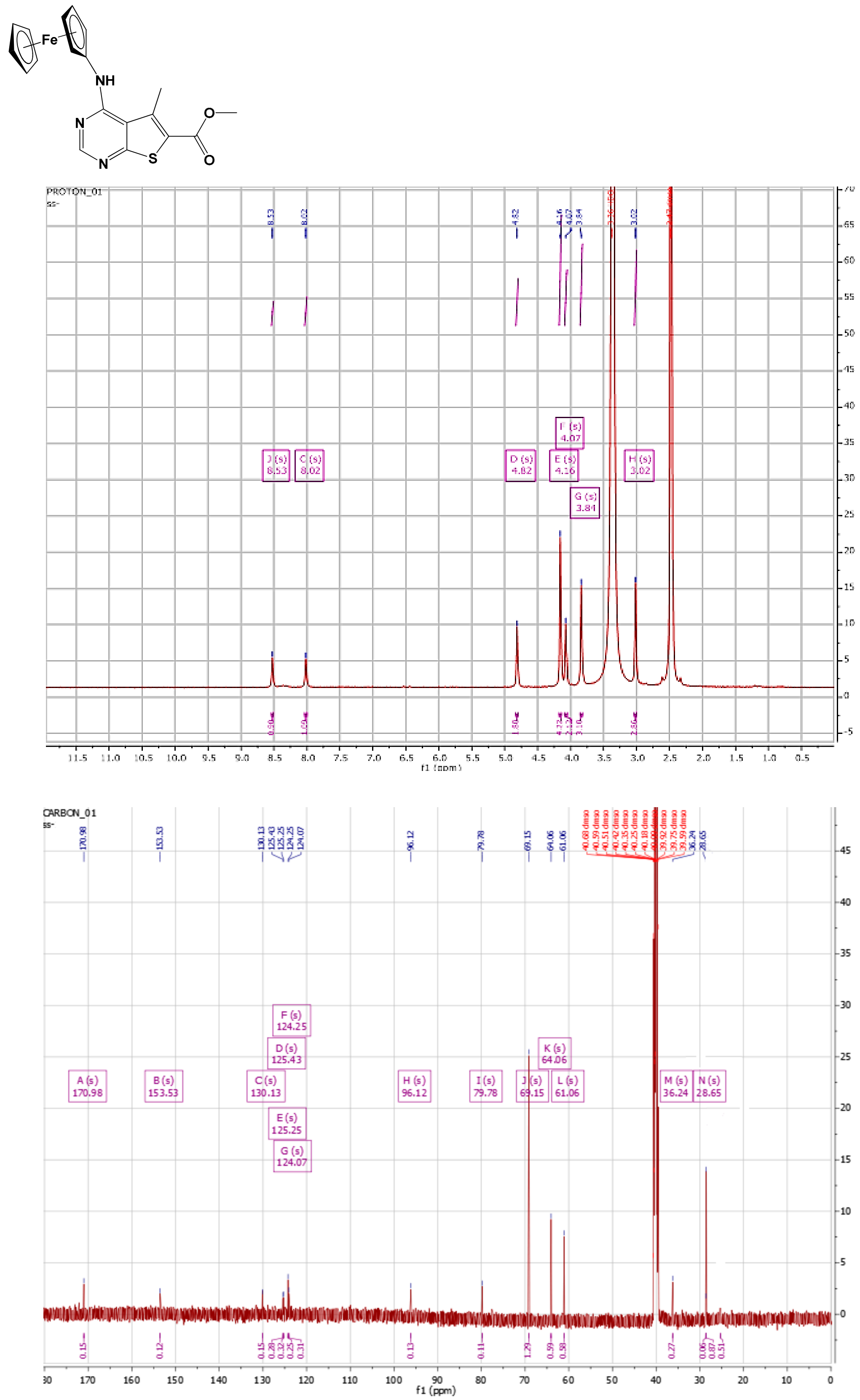

Figure S1. Scanned ^{1}H- and ^{13}C-NMR spectra of Methyl 5-methyl-4-(ferrocenylamino)thieno[2,3-d]pyrimidine-6-carboxylate **(2).**

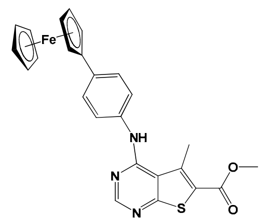

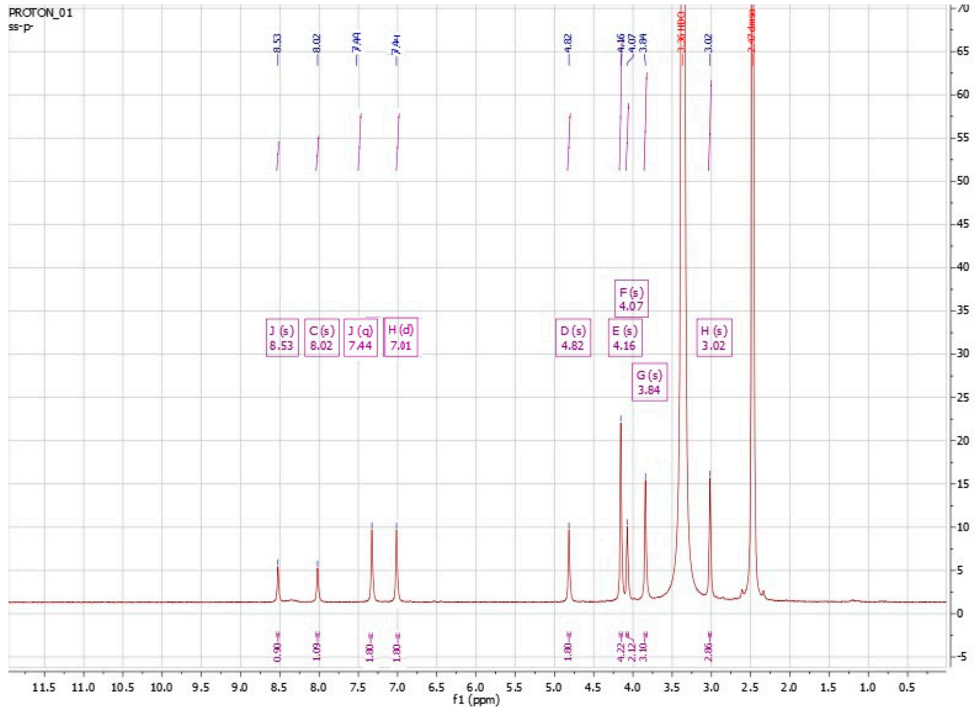

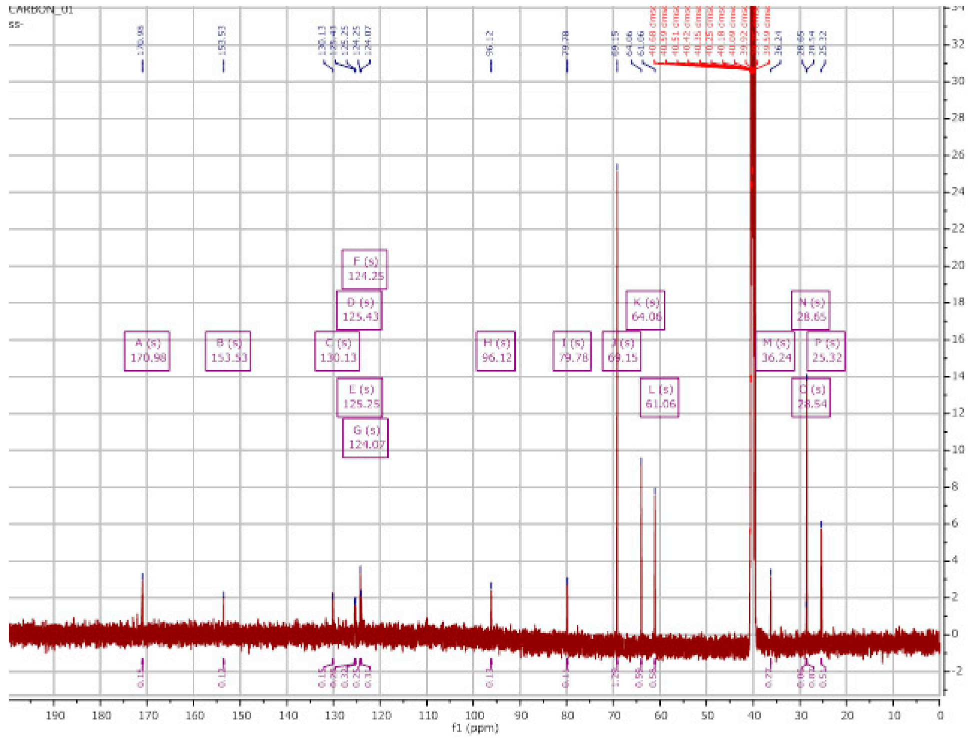

Figure S2. Scanned ^{1}H- and ^{13}C-NMR spectra of Methyl 4-(ferrocenyl-phenyl-4-ylamino)-5-methylthieno[2,3-d]pyrimidine-6-carboxylate (**4**).

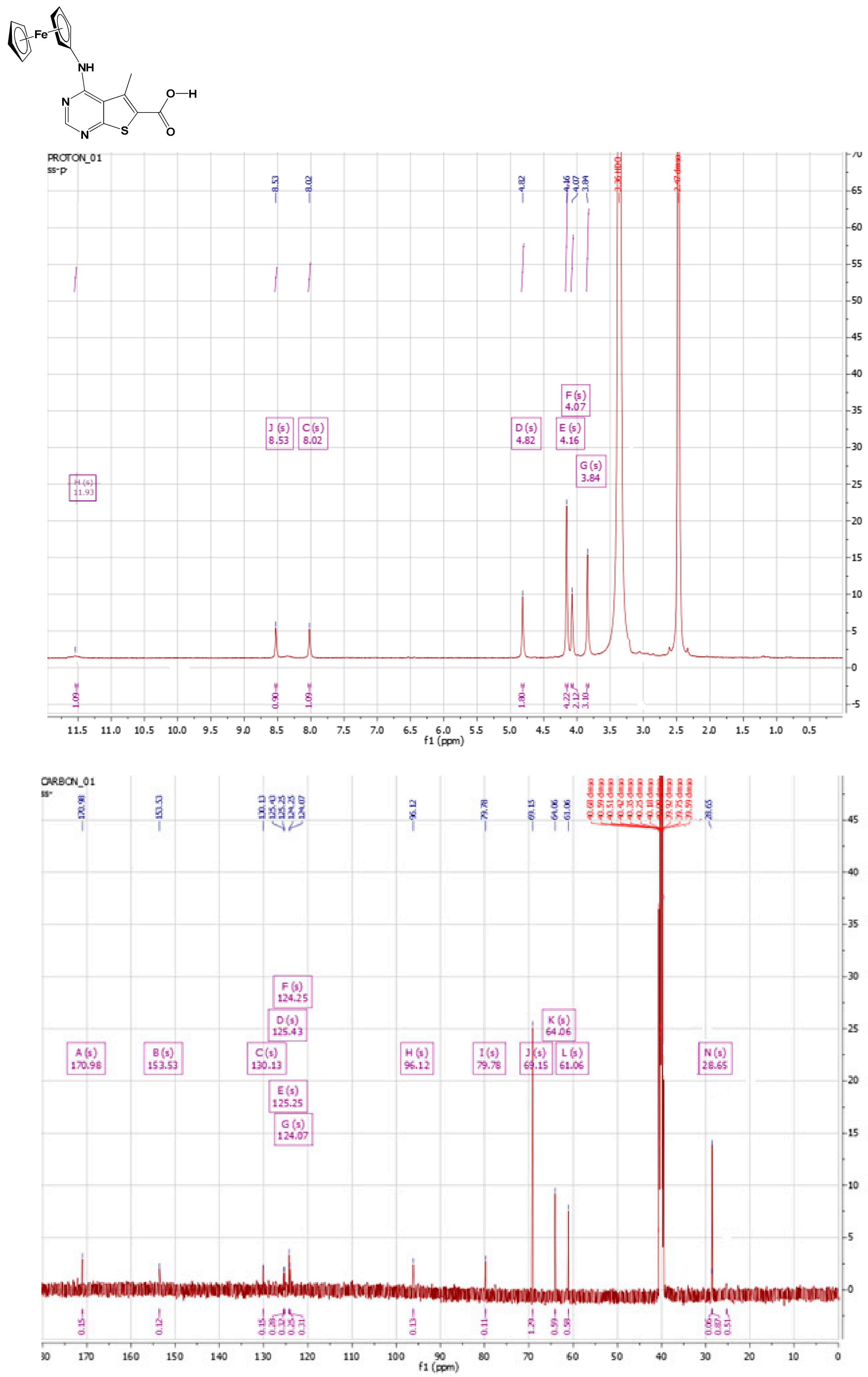

Figure S3. Scanned ^{1}H- and ^{13}C-NMR spectra of 5-methyl-4-(phenylamino)thieno[2,3-d]pyrimidine-6-carboxylic acid (**3**).

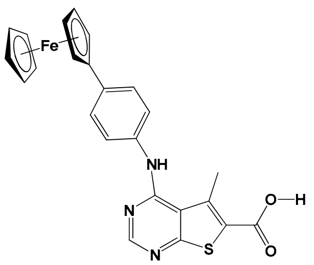

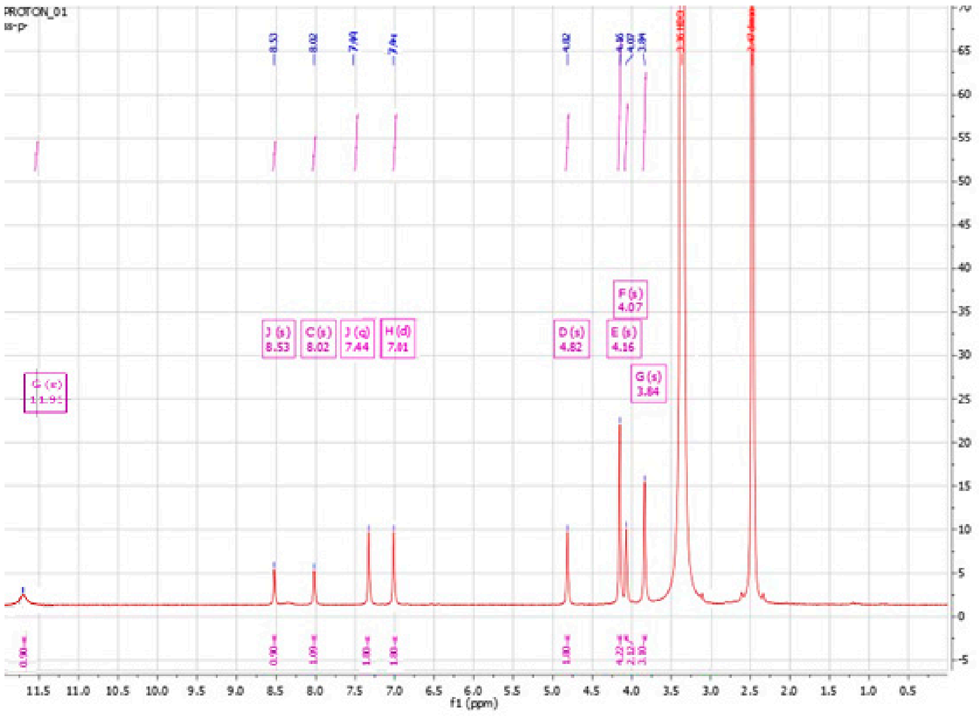

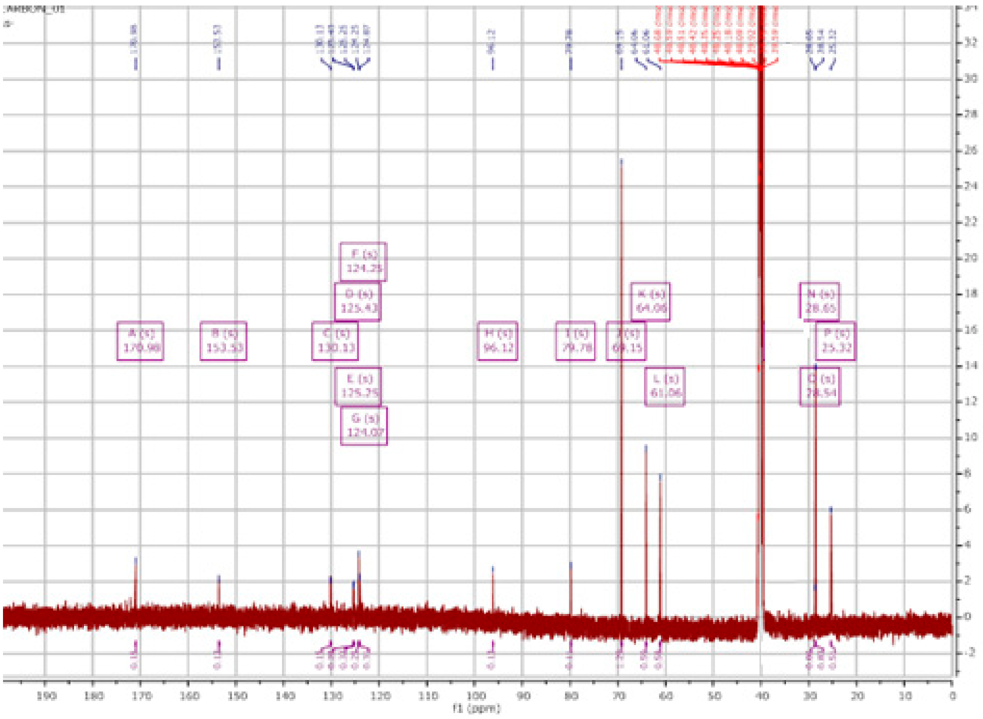

Figure S4. Scanned ^{1}H- and ^{13}C-NMR spectra of 4-(Ferrocenyl-phenyl-4-ylamino)-5-methylthieno[2,3-d]pyrimidine-6-carboxylic acid (**5**).

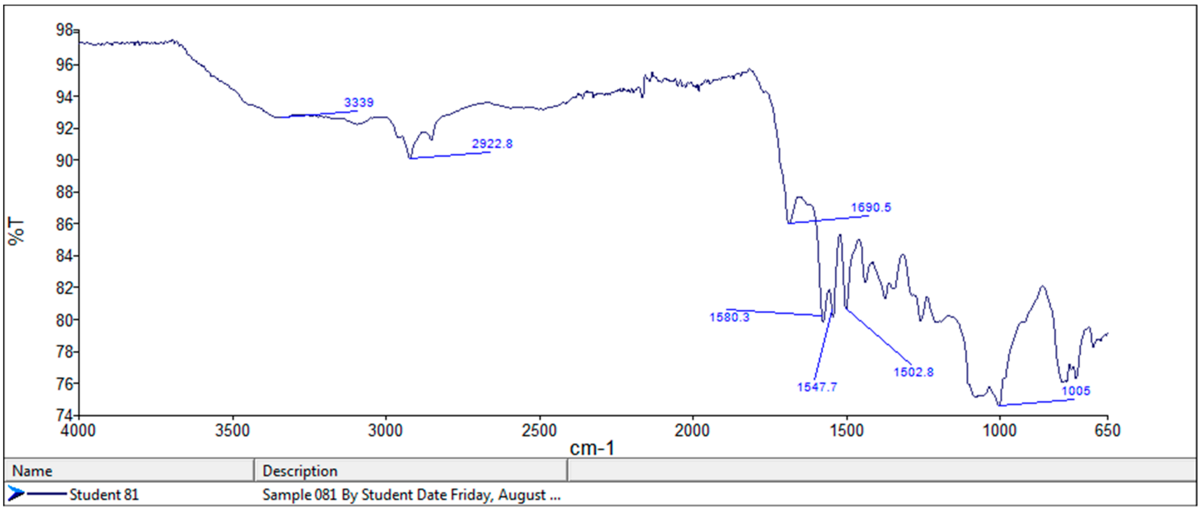

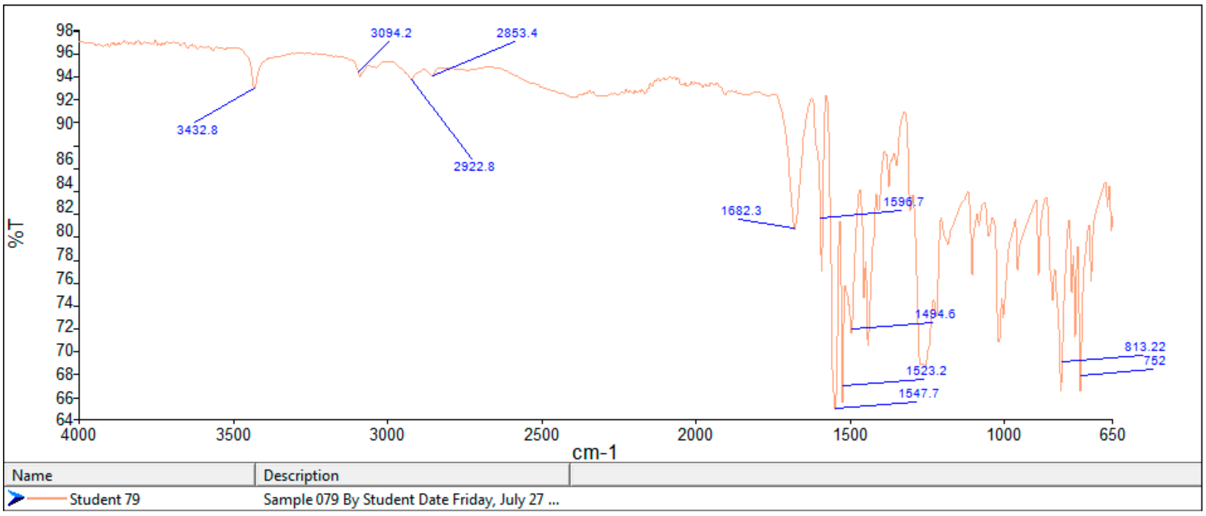

Figure S5. IR spectra of **3** and **5.**
